# Quantification of Cre-mediated recombination by a novel strategy reveals a stable extra-chromosomal deletion-circle in mice

**DOI:** 10.1186/1472-6750-8-18

**Published:** 2008-02-25

**Authors:** Wouter N Leonhard, Jeroen H Roelfsema, Irma S Lantinga-van Leeuwen, Martijn H Breuning, Dorien JM Peters

**Affiliations:** 1 Dept of Human Genetics, Center for Human and Clinical Genetics, Leiden University Medical Center, 2300 RC Leiden, The Netherlands; 2 National Institute of Public Health and the Environment (RIVM), Postbus 1. 3720BA Bilthoven, The Netherlands

## Abstract

**Background:**

Inducible conditional knockout animals are widely used to get insight in the function of genes and the pathogenesis of human diseases. These models frequently rely on Cre-mediated recombination of sequences flanked by Lox-P sites. To understand the consequences of gene disruption, it is essential to know the efficiency of the recombination process.

**Results:**

Here, we describe a modification of the multiplex ligation-dependent probe amplification (MLPA), called extension-MLPA (eMLPA), which enables quantification of relatively small differences in DNA that are a consequence of Cre-mediated recombination. eMLPA, here applied on an inducible *Pkd1 *conditional deletion mouse model, simultaneously measures both the reduction of the floxed allele and the increase of the deletion allele in a single reaction thereby minimizing any type of experimental variation. Interestingly, with this method we were also able to observe the presence of the excised DNA fragment. This extra-chromosomal deletion-circle was detectable up to 5 months after activation of Cre.

**Conclusion:**

eMLPA is a novel strategy which easily can be applied to measure the Cre-mediated recombination efficiency in each experimental case with high accuracy. In addition the fate of the deletion-circle can be followed simultaneously.

## Background

To study the effects of gene disruption in specific tissues throughout different stages of life, many inducible and conditional knock-out mouse models have been generated. The advantage of these models over conventional knock-out mice is that the gene of interest can be inactivated in the desired tissues at the desired moment, thereby bypassing embryonic lethality and undesirable side effects in other tissues. Conditional knockout systems frequently rely on the recombination of 'floxed' sequences. These are sequences that are flanked by two specific 34 base pair sequences, the so-called Lox-P sites [1-3]. Recombination is caused by the enzyme Cre, resulting in removal of the intervening sequence and one Lox-P site [1]. When the expression of Cre is under control of a tissue specific promoter and used in combination with an inducible system, such as the mutated estrogen receptor ligand-binding domain (ER^T2^), targeted genes can be inactivated in the desired tissues at different stages of life upon administration of for instance tamoxifen [4].

To understand the subsequent phenotype it is important to know the cell-type and the proportion of cells in which recombination occurred. Reporter models are very suitable to determine 'where' Cre is active, but do not always provide reliable information on the proportion of cells in which the target gene will be disrupted. In addition, to perform for instance gene expression experiments or to test therapeutic interventions, it is essential to know the exact proportion of cells in which recombination had occurred for each individual mouse. The quantification of the relatively small changes as a result of Cre-mediated recombination in a subset of cells is challenging and requires a highly quantitative technique. Although, after extensive optimization, quantitative PCR (qPCR) has been used successfully in our group to measure recombination efficiency in an inducible and conditional knock-out model [5,6], we sought for an alternative method that makes it possible to measure all variables in one reaction and thus yield a higher level of accuracy. For this reason, we designed a modified form of Multiplex Ligation-dependant Probe Amplification (MLPA), called extension-MLPA (eMLPA), with which we can easily quantify reduction of the floxed allele and increase of the deletion allele simultaneously, thereby minimizing the experimental variation.

The earlier described MLPA technique, commonly used in diagnostics to detect copy number variations in genomic DNA, uses probe-pairs that contain target specific and universal sequences [7]. These probe-pairs are hybridized immediately adjacent to each other onto the target DNA and after ligation, templates of unique sizes are generated. These templates contain universal sequences at both ends and can be amplified using a single primer pair. The strength of MLPA is that it enables the analysis of multiple genomic loci in a single reaction. In the case of conditional knock-out models however, the presence of a common Lox-P site impedes the simultaneous measurement of the floxed and deletion alleles. Adjustment of MLPA by an additional extension reaction, makes it possible to distinguish the different fragments and to quantify them in one reaction.

The principle of eMLPA was tested on a mouse model for Autosomal Dominant Polycystic Kidney Disease, containing the tamoxifen-inducible KspCad-Cre-ER^T2 ^construct, hereafter Cre, and the floxed *Pkd1 *allele (*Pkd1*^lox2-11^) [5,6]. In these mice Cre is expressed in the kidneys in the epithelial cells of all tubular segments of the nefrons. Upon administration of tamoxifen, Cre translocates from the cytoplasm to the nucleus and converts the *Pkd1*^lox2-11 ^allele, hereafter *Pkd1*^lox^, to the *Pkd1*^del2-11 ^allele. Using eMLPA, recombination efficiency was determined with high accuracy and reproducibility.

Additionally, the excised and circularized fragment that results from Cre-mediated recombination could also be detected, even five months after recombination was induced.

## Results

With the aim to get a more accurate measurement of Cre-mediated recombination in inducible and conditional knock-out mice, we have set up the extension MLPA (eMLPA) to quantify in one reaction, the mixture of alleles that are a result of Cre-mediated recombination. The earlier described MLPA technique is able to quantify multiple targets on a DNA template in a single reaction [7]. However, in the regular MLPA strategy, the probes will not be able to discriminate between the floxed and the deletion allele due to the presence of a common Lox-P site. In addition, since the Lox-P site is a palindrome, probes designed directly on the Lox-P site are likely to fail. Therefore, MLPA was modified in such a way that the alleles can be distinguished.

### eMLPA strategy

**Figure 1:**
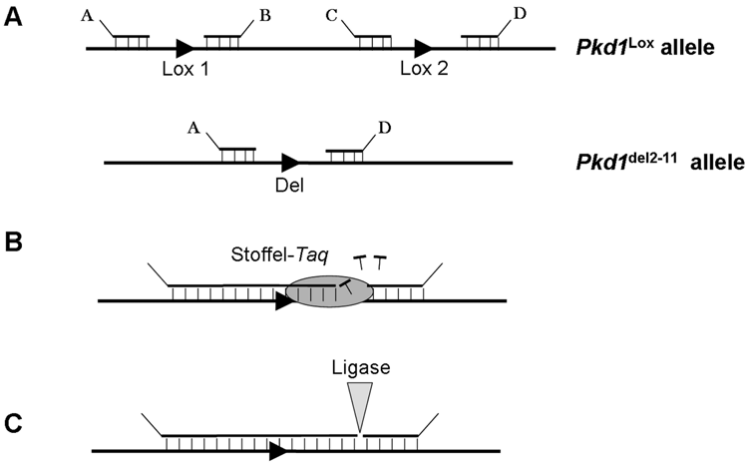
**The eMLPA strategy**. **(A) **The position of the probes A, B, C, and D in case of the *Pkd1*^lox ^allele or the *Pkd1*^del2-11 ^allele after hybridization. **(B) **The gap between probes is filled in using Stoffel-*Taq *Polymerase. **(C) **The remaining nick is ligated by ligase, completing the formation of the templates AB, CD, and AD which are amplified simultaneously in a PCR reaction containing a fluorescently labeled forward primer.

**Figure 2:**
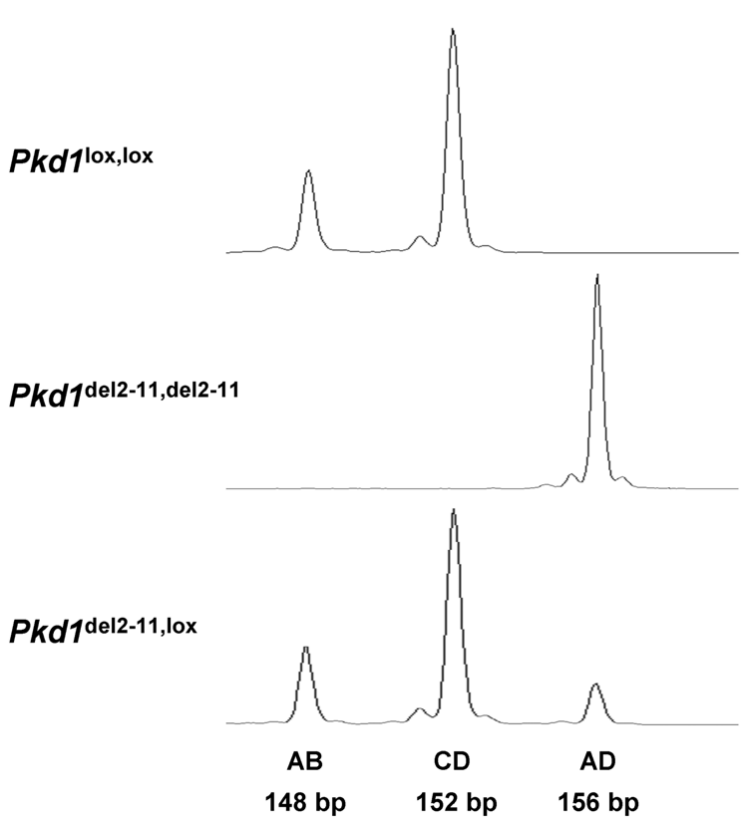
**The AB, CD and AD peaks obtained after capillary gel electrophoresis**. When only the *Pkd1*^lox ^allele is present two peaks, AB and CD, can be observed (Upper panel). A single peak, AD, is observed when only the *Pkd1*^del2-11 ^allele is present (Middle panel). When the *Pkd1*^lox ^and the *Pkd1*^del2-11 ^allele are both present, all three peaks can be observed (Lower panel).

The eMLPA strategy is outlined in Figure 1. Instead of designing the probes adjacent to each other, the probes were designed around the Lox-P sites. Probes A and B are located 46 bp from each other flanking the Lox-P site in intron 1 of the *Pkd1 *gene, and probes C and D are located 47 bp from each other flanking the Lox-P site in intron 11 (Figure 1). In regular MLPA the next step would be the ligation of the neighbouring probes to generate a template for PCR. In eMLPA however, a polymerase is required to fill in the gaps. Most DNA polymerases harbor besides 5'-3' polymerase activity, also 5'-3' exonuclease or strand-displacement activity. Since these activities will degrade or displace the right probe, no template for the PCR-reaction could be generated, leading to failure in the eMLPA strategy (not shown). The Stoffel fragment of *Taq*-polymerase has been reported to lack 5'-3' exonuclease or strand-displacement activities [8]. However, the optimum temperature of Stoffel-*Taq *is 72°C, which is slightly above the melting temperature of most of the probes. Unsurprisingly, no peaks were observed when the extension reaction was carried out at 72°C (not shown). Since the ligation reaction is performed at 54°C we combined the extension with the ligation reaction at that temperature, just by adding Stoffel-*Taq *and dNTP's to the ligation mixture. Already the addition of 0.1 U Stoffel-*Taq *and 2 mM of dNTP's to the ligation mixture resulted in strong peaks of the expected sizes after 29 rounds of amplification (Figure 2). The signals seem to increase somewhat up to 1 U of Stoffel-*Taq*, but did not change significantly when increasing amounts of Stoffel-*Taq *up to 10 U were added (not shown). To quantify the recombination efficiency in our inducible conditional mouse model we used 1 U Stoffel-*Taq *in the extension reaction.

### Quantification of recombination efficiency by eMLPA, in inducible and conditional *Pkd1 *mice

**Figure 3:**
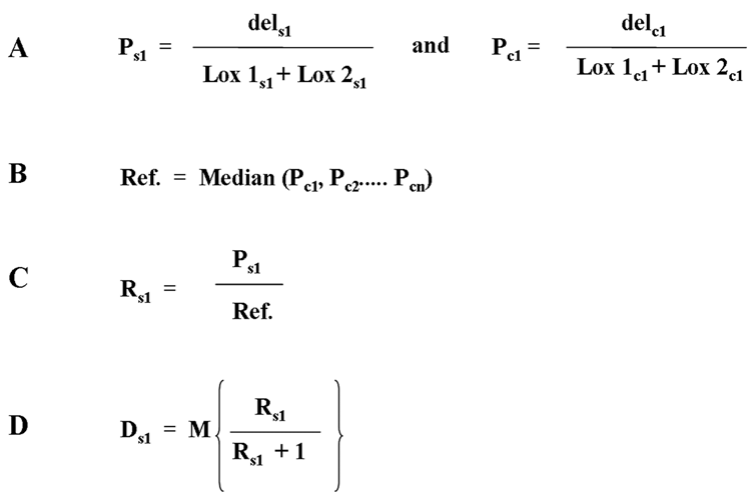
**Outline for calculating the percentage of deletion allele**. The percentage of *Pkd1*^del2-11 ^for a sample s1 is used as an example. **(A) **The peak-ratios (P) for all controls and sample s1 is calculated by dividing the height of the deletion peak with the sum of the lox peaks. **(B) **A fifty percent reference (Ref.) is calculated by taking the median of the peak-ratios from all controls. **(C) **The peak-ratio for sample 1 (P_s1_) is divided by and thus normalized to the fifty percent reference. The ratio of the *Pkd1*^del2-11 ^allele to the *Pkd1*^lox ^allele in sample 1 is, R_s1 _to 1. **(D) **Thus, the fraction of the *Pkd1*^del2-11 ^allele relative to the sum of the *Pkd1*^del2-11 ^and *Pkd1*^lox ^alleles is R_s1 _divided by R_s1 _plus 1. The total percentage of *Pkd1*^del2-11 ^in sample 1 (D_s1_) can now be calculated by multiplying this fraction with the maximum percentage (M) the *Pkd1*^del2-11 ^and *Pkd1*^lox ^alleles can have together. M is either 50% when initially one *Pkd1*^lox ^and one *Pkd1*^+ ^allele are present or a 100% when both alleles were either *Pkd1*^lox ^or *Pkd1*^del2-11^.

**Figure 4:**
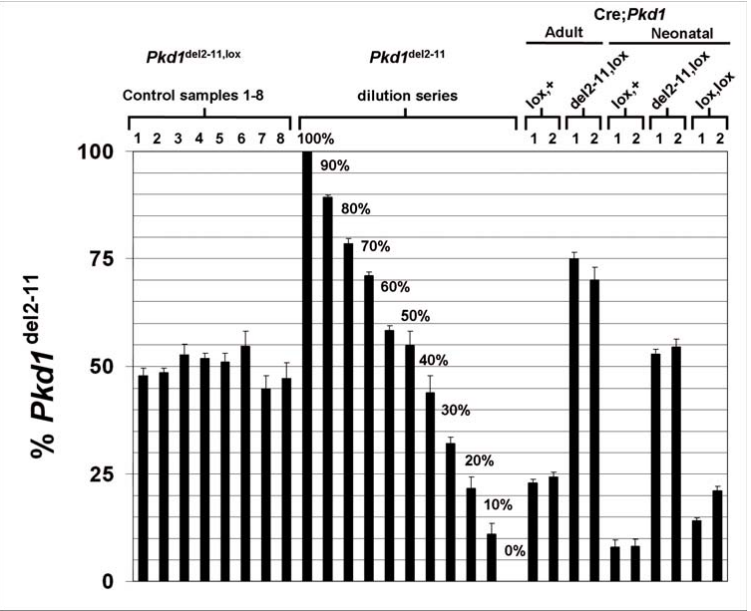
**Quantification of the percentage of *Pkd1*^del2-11 ^allele by eMLPA**. To validate the eMLPA strategy, the percentage of *Pkd1*^del2-11 ^allele is shown for eight *Pkd1*^del2-11, lox ^controls with an expected value of 50%, and for a dilution series ranging from a hundred to zero percent of *Pkd1*^del2-11 ^allele, obtained by diluting *Pkd1*^del2-11, del2-11 ^with *Pkd1*^lox, lox ^DNA at different ratios. All controls and samples from the dilution series gave the expected results within a five percent interval. The percentage of *Pkd1*^del2-11 ^allele is also measured in adult or neonatal tamoxifen treated Cre; *Pkd1*^lox,+^, Cre; *Pkd1*^del2-11, lox ^and Cre; *Pkd1*^lox, lox ^mice. The error bars represent the standard error of the mean. Hybridizations were performed three times and PCR's were performed in triplicate on each hybridization.

In mice having one or two *Pkd1*^lox ^alleles and the Cre transgene, deletion of the floxed gene can be induced in the kidneys by administration of Tamoxifen. If recombination occurs, the ratio of the peaks corresponding to the floxed and deletion alleles, changes. This ratio is fixed and should be stable among *Pkd1*^del2-11, lox ^mice lacking Cre. These mice carry one *Pkd1*-deletion allele in the germ-line [9], meaning that all cells contain one deletion allele. Therefore, in each experiment, we calculated the median of the ratios of eight *Pkd1*^del2-11, lox ^mice, which served as a fifty percent reference. To validate eMLPA, we included a dilution series in steps of ten percent ranging from a hundred to zero percent, by diluting DNA from embryonic mice with two *Pkd1*^del-11 ^alleles with DNA from mice containing two *Pkd1*^lox ^alleles. By relating the peak-ratios to the fifty percent reference, the *Pkd1*^del-11 ^percentages from each sample could be calculated (Figure 3). The measured percentages from all samples with a known proportion of *Pkd1*^del-11 ^allele resembled the expected values within a five percent interval (Figure 4). This demonstrates that eMLPA is well suited to quantify the amount of recombined allele ranging from zero to a hundred percent with high reproducibility.

In addition, samples from tamoxifen treated neonatal or adult Cre; *Pkd1*^del2-11, lox^, Cre; *Pkd1*^lox,+^, and Cre; *Pkd1*^lox, lox ^mice were analyzed. The results indicate that adult Cre; *Pkd1*^del2-11, lox ^and Cre; *Pkd1*^lox,+ ^mice that were treated with tamoxifen, gained 20–25% extra *Pkd1*^del2-11 ^allele, meaning that in 40–50% of renal cells recombination had occurred. Neonatal Cre; *Pkd1*^del2-11, lox ^and Cre; *Pkd1*^lox,+ ^mice that were treated with tamoxifen, gained approximately 3–8% extra *Pkd1*^del2-11 ^allele, indicating that in 6–16% of renal cells recombination had occurred. Neonatal Cre; *Pkd1*^lox, lox ^mice that were treated with tamoxifen, gained 14–21% extra *Pkd1*^del2-11 ^allele.

**Figure 5:**
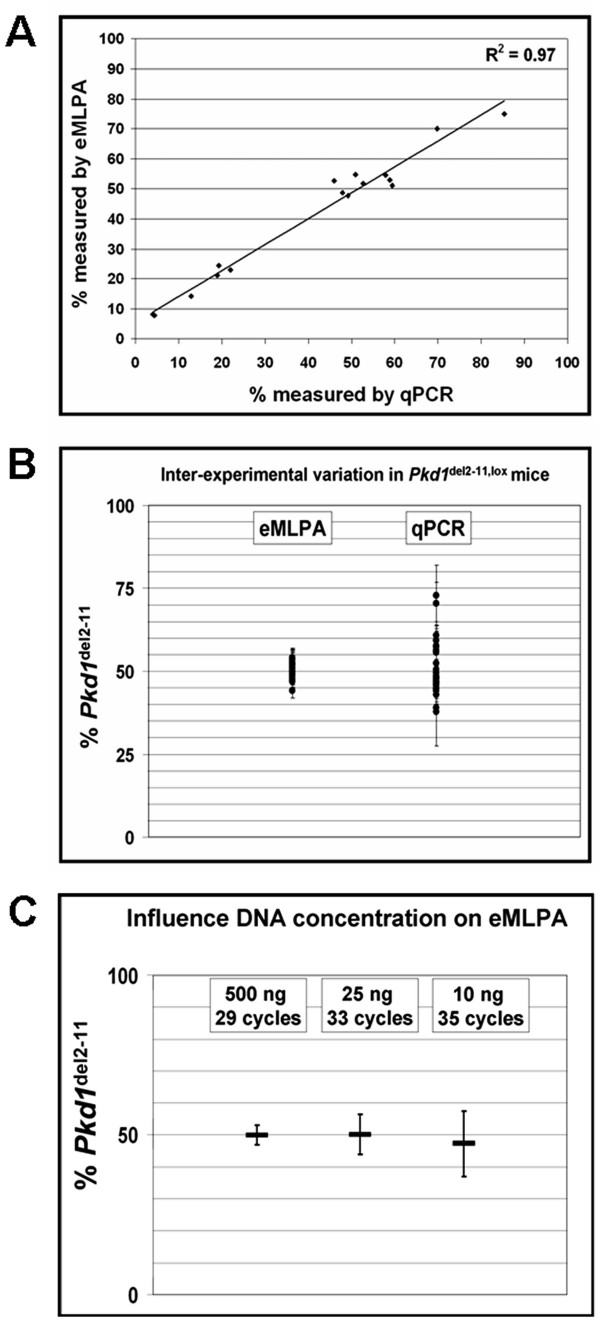
**Characterization of experimental errors in eMLPA and qPCR**. **(A) **Correlation of eMLPA and qPCR. To verify eMLPA, data obtained from samples analyzed with both qPCR and eMLPA are plotted against each other. **(B) **Inter-experimental variation in heterozygous control (*Pkd1*^del2-11, lox^) mice is presented. Raw data from five different eMLPA or qPCR experiments were combined. The percentage of *Pkd1 *deletion, which should be exactly 50%, was calculated relative to the median of all measurements. An eMLPA experiment consists of two or three hybridizations on which a single PCR was performed and a qPCR experiment consists of a triplo PCR. Each point shown is an average of these duplo or triplo measurements and the error bars represent the standard error of the mean. **(C) **The performance of eMLPA at low DNA concentrations. Single hybridizations were carried out on 500 ng, 25 ng or 10 ng of DNA from eight *Pkd1*^del2-11, lox ^mice, followed by extension, ligation and 29, 33 or 35 cycles of amplification respectively. *Pkd1*^del2-11 ^percentages were calculated relative to the median from the 500 ng hybridizations which was used as the 50% reference. From the eight *Pkd1*^del2-11, lox ^samples in each group; the 500 ng, 25 ng and the 10 ng hybridizations, the median and standard deviations from the *Pkd1*^del2-11 ^percentages are shown. Whereas the median from both groups at the lower DNA concentrations is close to the expected 50%, the variation in these groups is higher.

The data obtained by eMLPA are in line with the previous reported qPCR data (Figure 5A) [5,6]. However even after extensive optimization and selection of optimal primer sets out of at least six different primer combinations for both the deletion specific and the reference PCR, qPCR seemed to be more susceptible to random and systematic errors then eMLPA. In a typical experiment we now use two or three eMLPA hybridizations on which a single PCR is performed, while qPCR was performed three times in triplicate.

In this study, DNA from *Pkd1*^del2-11, lox ^mice was used as a fifty percent reference. However, when DNA from heterozygous deletion-mice is not available, an alternative reference should be established. For this purpose, a probe set has to be selected on a control locus, which can be any locus outside the recombination area, and should be used in addition to the probes for detection of the floxed allele. Similar to regular MLPA that is used to measure copy number variation, the peak-height of the control locus is related to the peak-heights of the floxed locus. The ratio between these peaks will then serve as a hundred percent reference in mice homozygous and as a fifty percent reference in mice heterozygous for the floxed allele. After recombination, the reduction of the floxed allele can be quantified by comparing the altered Lox-P/control peak-ratio with this reference.

### Characterization of random and systematic errors in qPCR and eMLPA

While random errors manifest themselves as large error bars, systematic errors will display a value significantly different from the true value. The latter may lead to false conclusions when samples with an unknown value need to be analyzed. To analyze potential variation, eMLPA and qPCR data from six *Pkd1*^del2-11, lox ^mice, which have exactly 50% *Pkd1*^del2-11 ^allele, were compared. The raw data from five different experiments were combined and the median was calculated. For the individual measurements, percentages were calculated relative to the median. The variation observed in qPCR was considerably larger compared to the variation seen in eMLPA (Figure 5B).

We further investigated the influence of lower DNA concentrations on the performance of eMLPA. Single hybridizations were carried out on 500 ng, 25 ng or 10 ng of DNA from eight *Pkd1*^del2-11, lox ^mice, followed by extension and ligation. From this reaction (in total 40 ul), 5 ul was used for PCR. Since 29 cycles of amplification was not sufficient to obtain considerable peaks at the lower DNA concentrations, the 25 ng and the 10 ng hybridizations were amplified for 33 and 35 cycles respectively. The *Pkd1*^del2-11 ^percentages were calculated using the median from the 500 ng hybridizations as the 50% reference. Next, the median and standard deviations were calculated from the eight *Pkd1*^del2-11, lox ^samples for each group (Figure 5C). The median obtained from the groups with the lower DNA concentrations were close to the expected 50%, indicating that peak-ratios are not significantly influenced by lower DNA concentrations combined with more cycles of amplification as described previously [10]. Although more variation is seen at lower DNA concentrations, a single PCR on a single hybridization on only 10 ng of DNA, can already give an estimation of the recombination efficiency. More accurate results at low DNA concentrations may be obtained when more PCR reactions are performed on the same hybridization.

### Detection of the circular extra-chromosomal deletion product of Cre

**Figure 6:**
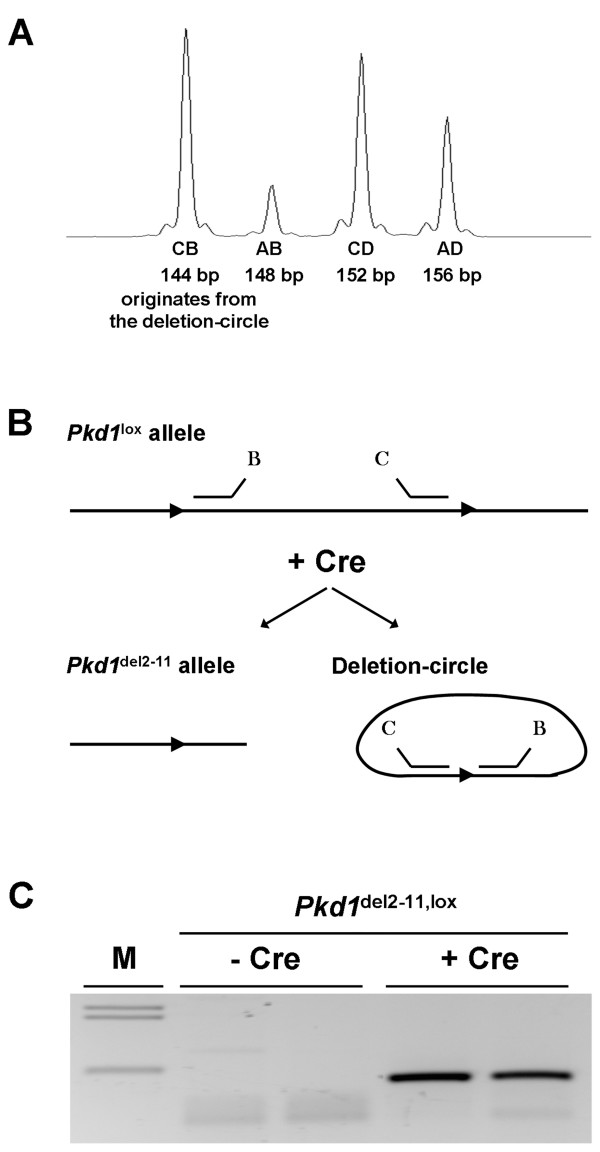
**Detection of the deletion-circle as a result of Cre-mediated recombination**. **(A) **An example of the four observed peaks in an adult tamoxifen treated Cre; *Pkd1*^del2-11, lox ^mouse, three peaks corresponding to the *Pkd1*^lox ^and *Pkd1*^del2-11 ^alleles plus an additional 144 bp peak. **(B) **The possible locations of probes B and C on the *Pkd1*^lox ^allele and on the deletion-circle. Probes B and C can only form a product when the floxed fragment is excised and circularized by Cre. **(C) **Hybridization of probes B and C followed by extension, ligation, 36 cycles of amplification and agarose-gel-electrophoresis does not result in a product when DNA from two *Pkd1*^del2-11, lox ^mice was used, but when DNA from kidneys from two adult tamoxifen treated Cre; *Pkd1*^del2-11, lox ^mice was used as a template, a distinct product of 144 bp was observed.

Beside the peaks corresponding to the *Pkd1*^lox ^and *Pkd1*^del2-11 ^alleles, tamoxifen induced Ksp-CreER^T2 ^mice having one or two *Pkd1*^lox ^alleles showed an additional 144 bp peak, of variable height (Figure 6A). In mice that were left untreated or lacked the Ksp-CreER^T2 ^transgene this peak was never observed. We investigated the possibility that this extra peak originated from the circular extra-chromosomal deletion product, hereafter the deletion-circle, of the recombination process [1]. Only when this excised floxed DNA fragment is present in a circular configuration (Figure 6B) probes B and C potentially can form a 144 bp product. Indeed, when eMLPA was carried out using just probes B and C, only in the samples in which recombination occurred, a clear product was detected after agarose-gel-electrophoresis (Figure 6C). To confirm that this product originated from the extra-chromosomal deletion-circle, sequence analysis in both directions of the B to C product was carried out. The B to C sequence exactly matched the sequence that would be expected from the floxed DNA fragment when excised and circularized by Cre-recombinase.

## Discussion

To understand the function of genes inactivated by Cre-mediated recombination and the resulting phenotypes, it is essential to know the exact location as well as the efficiency of the recombination events. This can be achieved by histological analysis of the target protein by antibody staining but will only be possible when an excellent antibody is available and the expression is high enough. The generation of reporter models, capable of visualizing Cre activity by expressing fluorescent proteins, or proteins that can be easily detected via enzymatic reactions, allows the localization of active Cre but may not provide reliable knowledge about the recombination efficiency in each individual case, due to variability in the efficiency of Cre between different floxed loci, induction protocols, developmental stages, genetic backgrounds or even between littermates [5,6,11,12]. However, once the location has been established, alternative methods may be used to determine the recombination efficiency of the target locus in individual mice.

Since Cre-mediated recombination may result in the conversion of a floxed allele to a deletion allele only in a subset of cells, the changes to be measured are usually subtle and thus require a highly quantitative and robust technique. To quantify Cre-mediated recombination, southern and western blotting for example can be used but require a relatively high amount of DNA or protein, are labor-intensive and will not be able to detect small differences with sufficient reliability. More commonly nowadays, qPCR has been applied, a method that is generally used to detect two or more fold differences. We successfully applied qPCR to measure the percentage of *Pkd1*^del2-11 ^allele [5,6]. However, despite the use of high-quality DNA as assessed by nanodrop measurements and extensive optimizations, qPCR seemed to be susceptible to random and systematic errors. Presumably, since signals from different reactions, a reference PCR and a deletion specific PCR, are compared to each other. In this paper we describe and validate the eMLPA technique, a novel strategy which can be used in a 96-well format and measures all parameters in a single reaction, allowing all possible information within a sample to be retrieved and experimental variation to be minimized.

Although regular MLPA is also able to measure multiple loci in one reaction, it cannot measure the floxed and the deletion allele simultaneously, due to the presence of a common Lox-P site. Furthermore, when the extra-chromosomal deletion-circle is still present, MLPA will not be able to distinguish this fragment from the floxed allele, making it impossible to measure the reduction of the floxed allele. The results obtained by eMLPA will not be affected by the deletion-circle, provided that the different fragment sizes do not overlap with each other. Probes should therefore be selected in such a way that all loci to be tested, including the possible deletion-circle, will result in peaks of different sizes. With eMLPA we could reproducibly quantify the recombination efficiency in our *Pkd1 *conditional knockout model using 300 to 500 ng of DNA. However, also at lower DNA concentrations of 10 to 25 ng per hybridization, especially relevant when working with small specimens, valuable information on the recombination efficiency can be obtained by increasing the amount of cycles of amplification and perform more PCR reactions on a single hybridization. Although, as described previously [10] relative signal intensities between peaks did not change significantly by varying the amount of cycles in the PCR and the inter-experimental variation in eMLPA is considerably smaller compared with qPCR, we recommend including a set of six to eight control samples in each experiment to obtain a reliable reference.

We observed differences in recombination efficiencies between adult and newborn mice. Since newborn mice received tamoxifen in the milk via weaning mothers and adult mice directly through a feeding needle, adult mice may receive higher concentrations of tamoxifen. In addition we also measured two-fold higher Cre-expression in adult mice [6]. Other explanations however, like differences in tissue composition, cannot be ruled out [6].

Using eMLPA, we could clearly detect the deletion-circle, even five months after activation of Cre by tamoxifen. In *Pkd1*^del2-11, lox ^mice, carrying the deletion allele in the germ line and lacking Cre, this fragment was not detected. Since the deletion-circle lacks an origin of replication it is unlikely that it will be replicated during cell-division. However, in non-dividing cells, the deletion-circle apparently can be stable for a long time without being degraded, a phenomenon which also has been seen in plants and in *Drosophila *but, to the best of our knowledge, has not yet been reported for mouse models [13,14]. Even more, the *Drosophila white *gene on the extra-chromosomal circle was expressed in the eyes during a substantial period of time and the expression could increase after excision [14]. Therefore, when larger DNA fragments are excised containing entire genes that potentially could be transcribed, it may be desirable to follow the fate of the excised fragment. We observed variability in the height of the peak that corresponded to the deletion-circle. This may be explained by the type of DNA-isolation protocol that we used, designed to isolate high-molecular genomic DNA, but could also result from variation in proliferation between different mice. Upon isolation of total DNA, it should be possible to simultaneously quantify the recombination efficiency and the amount of circular excised fragment using eMLPA.

In addition, to the *Pkd1 *inducible and conditional knock-out model, eMLPA was used to measure recombination efficiency of the *Fc gamma RII *gene, in a *B*-cell specific inducible Cre mouse-model. In this model the recombination efficiency in spleen, measured by eMLPA, resembled the level of *Fc gamma RII *protein in *B*-cells as quantified by FACS-analysis. Even more, also in these samples the deletion circle was clearly detectable (Borros *et al*. manuscript in preparation).

## Conclusion

eMLPA is a novel method, set up to measure Cre-mediated recombination, which has the advantage to measure gene disruption in a relatively small fraction of cells with high accuracy. Additionally, the fate of the excised DNA fragment can be followed simultaneously. eMLPA can be applied on a large number of samples, is easy to set up and requires equipment present in most labs.

## Methods

### Sample preparation

Tamoxifen treatment has been described previously [5]. Adult mice, 3–4 months of age, have been treated with 5 mg tamoxifen for 3 consecutive days, using a feeding needle. Weaning mothers, 3–6 months old, received a similar treatment starting at postnatal day 4 (PN4) of the progeny, and these newborn mice received tamoxifen via breast-feeding from the mother. Mice were sacrificed one to five months after tamoxifen administration and kidneys were collected and snap-frozen in liquid nitrogen from *Pkd1*^del2-11, lox^, *Pkd1*^lox, lox ^or *Pkd1*^lox,+ ^mice, treated or untreated with tamoxifen, depending on the presence or absence of the KspCad-Cre-ER^T2 ^transgene. Also 14-day-old embryos were used in order to obtain *Pkd1*^del2-11, del2-11 ^material. To simultaneously isolate DNA as well as total RNA, for possible future gene expression analysis, kidneys were homogenized in 500 μl 1% 2-mercaptoethanol in PBS, using the magNA lyser (Roche). To isolate total RNA, 750 μl Tri-Reagent (Sigma-Aldrich The Netherlands) was added to 250 μl of the homogenate, after which the manufacturers' protocol was further followed. The remaining part of the homogenate was used to isolate DNA using AX-G100 columns (Bioké, Leiden, The Netherlands) according to the manufacturers' protocol. From a minority of samples we only isolated DNA. These samples were homogenized in Buffer G2 (Bioké, Leiden, The Netherlands) with an ultra-thurax and DNA was isolated as described above.

All experiments using mice were approved by the local animal experimental committee of the Leiden University Medical Center and by the Commission Biotechnology in Animals of the Dutch Ministry of Agriculture.

### Probe design

Two probes, A and B, were designed to detect the Lox-P site in intron 1 of the *Pkd1 *gene (Lox 1) and two other probes, C and D, to detect the Lox-P site in intron 11 (Lox 2.) (Figure 1A). In addition to the target specific sequence, the left probe from each probe pair contains a 19 nt sequence on its 5' end, similar to the sequence of the labeled forward primer used in the PCR and the right probe from each pair contains the target sequence for the reverse primer on its 3' end. The primers used are the MLPA-primers described by Schouten *et al*. [7]. Furthermore, all probes were designed to have a melting temperature above 69°C, a GC content between 35 and 60 percent (determined by free to download software: Raw-probe, from MRC-Holland; Amsterdam, The Netherlands) and the right probes were phosphorylated on their 5' end. All probes were obtained from BioLegio (Nijmegen, the Netherlands) and sequences are available upon request. The product AD will be formed when the *Pkd1*^del2-11 ^allele is present. The products AB, CD and AD, differ four nucleotides in length so that they can be distinguished when run over a capillary sequencer. Due to the palindromic sequence of the Lox-P site, probes were designed around these sequences leaving a gap between the left and right probe that can be filled in during the extension reaction. This process is outlined in Figure 1B and 1C.

### eMLPA reaction

5 μl of 60–100 ng/μl DNA samples were heated at 98°C for 5 minutes and then placed on ice. Hybridizations were performed by adding 1.5 μl MLPA buffer (MRC-Holland; Amsterdam, The Netherlands) and 1.5 μl probe mix, containing a probe concentration of 4 nM, heating the samples at 95°C for 5 minutes followed by a 4 hour hybridization step at 60°C.

The extension and ligation reactions were performed simultaneously by adding 2 mM dNTP's and 1 U Stoffel *Taq *polymerase (Applied Biosystems) for the extension, and 3 μl Buffer A, 3 μl buffer B, 1 μl Ligase-65 (MRC-Holland) for the ligation, to a total volume of 40 μl while the samples and the extension/ligation mixture were pre-incubated at 54°C. The reactions were performed at 54°C for 25 minutes followed by the inactivation of the ligase at 95°C for 5 minutes. 5 μl of the samples was used in a 25 μl PCR reaction containing 1 μl primer-mix and 1 U Salsa polymerase (MRC-Holland) in 45 mM Tris-HCl pH8.5, 11 mM (NH_4_)_2_SO_4_, 4.5 mM MgCl_2_, 5 μM EDTA, 0.5 mM dNTP's and 0.11 mg/ml Bovine serum albumin (Fraction 5). The PCR reaction consisted of 29 rounds of amplification (30 s at 95°C, 30 s at 60°C, 45 s at 72°C). 2 μl of the amplified samples were mixed with 10 μl Hi-Di Formamide containing GeneScan-500 ROX size standard (Applied Biosystems) and run on a 3730 DNA analyzer (Applied Biosystems).

### eMLPA data analysis

For data analysis GeneScan analysis 3.5 and Microsoft Excel software were used.

The samples (s1, s2....sn) in each experiment consisted of DNA from kidneys from Cre; *Pkd1*^del2-11, lox ^or Cre; *Pkd1*^lox,+ ^mice in which an unknown amount of the *Pkd1*^lox ^allele has been converted to the *Pkd1*^del2-11 ^allele. To calculate the total percentage of *Pkd1*^del2-11 ^allele in these samples, a fifty percent reference was used. This reference was calculated from controls (c1, c2....cn), consisting of DNA from kidneys from *Pkd1*^del2-11, lox ^mice, having a fixed amount of 50% deletion allele.

First, the peak-ratios (P) were calculated for all samples and controls by dividing the height of the deletion peak by the sum of the heights of the two lox peaks, resulting in (P_s1_, P_s2_.... P_sn_) for the samples and (P_c1_, P_c2_.... P_cn_) for the control samples (Figure 3A).

Since the controls harbor exactly fifty percent *Pkd1*^del2-11 ^allele, the peak-ratios found in the controls represent fifty percent *Pkd1*^del2-11 ^allele. To calculate a robust fifty percent reference (Ref.), the median was obtained from the peak-ratios of the controls (Figure 3B).

All peak-ratios could then be normalized by dividing the peak-ratios with the fifty percent reference, resulting in the actual *Pkd1*^del2-11^ to *Pkd1*^lox ^ratio (R) (Figure 3C). Ideally, all controls should have an R-value of one, representing a *Pkd1*^del2-11 ^to *Pkd1*^lox ^ratio of 1:1.

From this ratio, the total percentage of *Pkd1*^del2-11 ^(D) could be calculated by dividing the *Pkd1*^del2-11 ^fraction (R) with the sum of the *Pkd1*^del2-11 ^and *Pkd1*^lox ^fractions (R + 1) which is then multiplied by the maximum percentage (M) of the *Pkd1*^del2-11 ^and *Pkd1*^lox ^alleles together (Figure 3D). When both alleles are either *Pkd1*^del2-11 ^or *Pkd1*^lox^, M is a hundred percent and when one wild-type allele is present, M is fifty percent.

### Quantitative PCR

qPCR was performed as described previously [5,6]. The *Pkd1*^del2-11 ^specific PCR was carried out using primers that were selected in intron 1 and intron 11 flanking the deletion, and compared to a reference PCR in which a fragment of exon 36 of the undeleted distal part of the *Pkd1 *gene was amplified. DNA isolated from *Pkd1*^del2-11, lox ^mice, containing 50% *Pkd1*^del2-11 ^allele, was used as a 50% reference. All measurements were performed three times in triplicate.

## Authors' contributions

WNL contributed to the conceptual idea, carried out the experiments and drafted the manuscript. JHR contributed to the conceptual idea of the eMLPA. ISLL provided the animal model on which the study was performed. MHB participated in coordination. DJMP participated in coordination, discussion and helped writing the manuscript. All authors have read and approved the final manuscript.
